# Influence of the Acceptor Fluid on the Bupivacaine Release from the Prospective Intra-Articular Methylcellulose Hydrogel

**DOI:** 10.3390/pharmaceutics16070867

**Published:** 2024-06-27

**Authors:** Dorota Wójcik-Pastuszka, Anna Frąk, Witold Musiał

**Affiliations:** Department of Physical Chemistry and Biophysics, Faculty of Pharmacy, Wroclaw Medical University, ul. Borowska 211A, 55-556 Wrocław, Poland; dorota.wojcik-pastuszka@umw.edu.pl (D.W.-P.); annafrak98@gmail.com (A.F.)

**Keywords:** bupivacaine, intra-articular hydrogel, sodium hyaluronate, release, kinetics, FTIR study, DSC study

## Abstract

Injections are one way of delivering drugs directly to the joint capsule. Employing this possibility, local anesthetic, such as bupivacaine (Bu), in the form of the suspension can be administered. The aim of this work was to propose a methylcellulose-based hydrogel-incorporated bupivacaine for intra-articular injections and to study the release kinetics of the drug from the hydrogel to different acceptor media, reflecting the synovial fluid of a healthy joint and the synovial fluid of an inflamed joint. The drug release studies were performed employing the flow apparatus. The drug was released to four different acceptor fluids: phosphate buffer pH = 7.4 (PBS7.4), phosphate buffer pH = 6.8 (PBS6.8), phosphate buffer pH = 7.4 with the high-molecular-weight sodium hyaluronate (PBS7.4H), and phosphate buffer pH = 6.8 with the low-molecular-weight sodium hyaluronate (PBS6.8L). The investigation was carried out at the temperature of 37 °C. The absorbance of the Bu released was measured at the wavelength of 262 nm every 2 min for 24 h. The release profiles of Bu to the acceptor media PBS7.4, PBS6.8, PBS7.4H, and PBS6.8L were described best by the first-order kinetics and the second-order equation. According to these models, the release rate constants were the highest when Bu was released to the fluid PBS7.4 and were k_1_ = (7.20 ± 0.01) × 10^−5^ min^−1^ and k_2_ = (3.00 ± 0.04) × 10^−6^ mg^−1^ × min^−1^, respectively. The relative viscosity of the acceptor medium, its pH, and the addition of high-molecular-weight or low-molecular-weight sodium hyaluronate (HAH or HAL) to the acceptor fluid influenced the drug dissolution. The release of Bu into the medium reflecting healthy synovial fluid takes a different pattern from its release into the fluid of an inflamed joint.

## 1. Introduction

Intra-articular injections are applied for local drug delivery in the treatment of inflammatory joint conditions. The joint surfaces, enclosed within a joint cavity, are lined with hyaline cartilage. The cartilage of the joint surface and the high-viscosity synovial fluid filling the joint capsule ensure the smooth, almost frictionless movement of the bones [1,2]. The required viscosity of the synovial fluid is provided by a high-molecular-weight hyaluronic acid or its sodium salt (HA). HA is constantly secreted into the joint and removed by the synovium; HA enables the protection of the articular cartilage and the delivery of nutrients to it. The HA concentration in healthy synovial fluid achieves 2.2 ± 1.6 mg [3]. The pH of healthy synovial fluid is usually between 7.2 and 7.4. In the case of inflammation, the pH decreases and may reach values 6.8 to 7.1 [4]. It was found that, after the initiation of joint sepsis in horses, the pH decreases below 6.9 within 12 to 24 h [5]. The acidic environment during joint disorders stimulates chondrocytes and synoviocytes to secrete cytokines (IL-1β). This causes the expansion of the subsynovial capillaries, increasing their permeability and, for example, causing the penetration of inflammatory cells into the joint space that are responsible for the inflammatory effect [4,5,6,7]. Under these conditions, high-molecular-weight HA can be degraded by enzymes released from the inflammatory tissues or bacteria or by oxidative stress. Chains composed of 25–50 disaccharide units have inflammatory, immune-system-stimulating, and angiogenic effects [7]. According to Ferreira et al. [8], the low-molecular-weight HA, after cell damage or under stress conditions, activates the immune response. Fragments coming from the high-molecular-weight HA decomposition play the role of pro-inflammatory markers, activate and promote cell maturation, and increase the release of pro-inflammatory cytokines, such as interleukin 1 beta (IL-1β), as well as tumor necrosis factor α (TNF-α). Additionally, the low-molecular-weight HA accelerates the expression of chemokines and cell proliferation. The interaction of low-molecular-weight HA with receptors, such as TLRs 2 and 4, also evokes a pro-inflammatory effect. However, the high-molecular-weight HA, present in healthy cells, decreases the release of cytokines as well as phagocytosis by macrophages. It plays an anti-inflammatory role resulting in the interaction with the CD44 receptor.

The viscoelastic properties of synovial fluid in the inflamed joint are modified. As a result of the HA decomposition, the low-molecular-weight HA appears, which reduces the viscosity of the synovial fluid [7,9,10].

**Figure 1:**
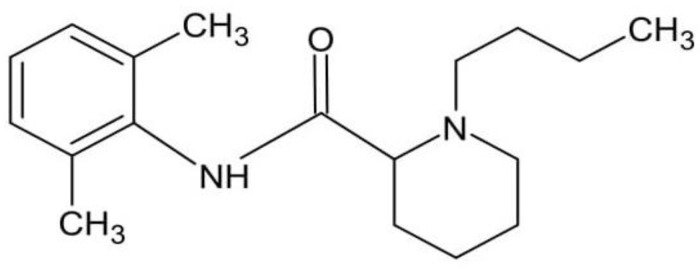
The structure of bupivacaine.

Bupivacaine (Bu) belongs to the class of amino–amide local anesthetics. According to Shah et al. [11] and Leone et al. [12], a local anesthetic agent should possess the following: sufficient duration of action, low toxicity, short onset time, high solubility in water, and a feasible sterilization possibility; these characteristics result from structural aspects of the molecule. The Bu is composed of three units: an aromatic group; an intermediate chain, which includes an amide link; and an amine group (Figure 1). Weinberg et al. [13] also found the connection between the structure of Bu and its solubility in polar and non-polar solvents, pharmacokinetics, and clinical effects. The lipophilicity of the aromatic ring ensures membrane permeation, and influences the intrinsic potency and onset time in the central compartment. The hydrolysis of Bu results in amide bond decomposition. Bu molecules are biodegraded mainly in the liver [14].

Bu is a long-acting drug used in surgery to reduce postoperative pain, when oral painkillers are not administered, and enables a reduction in their side effects [15,16,17]. Bu is applied for infiltration anesthesia, nerve blocks, epidural and intra-articular anesthesia [18], and in various diseases of the musculoskeletal system [11].

Bu is available and marketed as an injectable 0.25% or 0.5% saline solution. Fox et al. [19] revealed that 0.5% as well as 0.25% Bu provided pain relief in 5–10 min after the injection, throughout 1 week. The 0.5% Bu solution provided better pain relief compared to the 0.25% Bu solution. Injections into the joint space may increase the bioavailability of the drug substance, may reduce the number of adverse reactions, and may decrease the costs of therapy. However, the drugs are quickly eliminated from the joint capsule, and the residence time of the injected materials is short [2]. Many attempts have been made to increase the duration of the local anesthetic action. Hydrogels were proposed as a promising dosage form for numerous drugs, due to their capability of controlling the drug release pattern [20].

**Figure 2:**
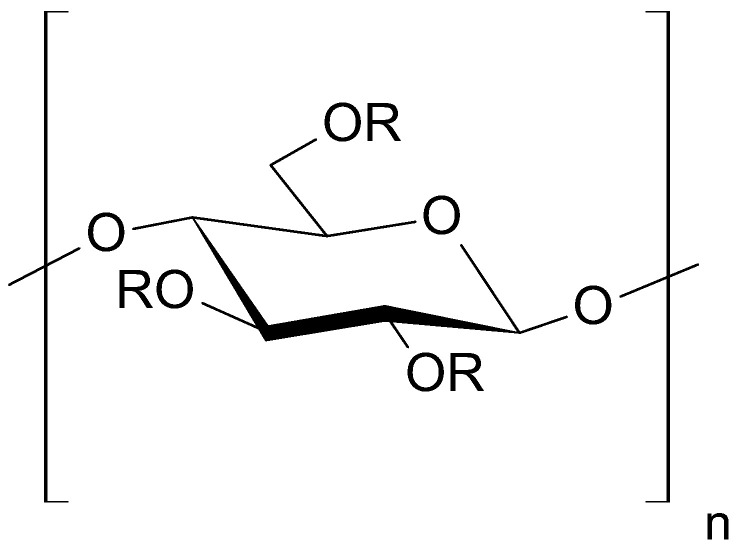
The structure of methylcellulose (MC); R = H or CH_3_.

Gels consist of a gelling substance dispersed in a proper medium. In hydrogels, water is applied as the dispersant, whereas the polymer is the gelling agent [21]. A hydrogel is composed of hydrophilic polymer chains that form a three-dimensional network, which enables the absorption of high amounts of water. Eventually the polymer swells, retaining the absorbed water or biological fluids entrapped in the polymeric network. Different functional groups such as amino (–NH_2_), carboxylic (–COOH), and sulfonate (–SO_3_H) are responsible for the formation of the polymeric network [22]. Methylcellulose (MC) belongs to the synthetic non-ionic polymers group, and forms stable, transparent, highly viscous gels (Figure 2) [20,23,24,25,26].

The anomalous behavior of MC-based hydrogel and its biocompatibility make it a promising matrix for injectable systems that may exhibit a rapid increase in viscosity at body temperature [27]. Hydrogels for intra-articular injections are of great interest, as they may prolong the therapeutic effect [28]. Moreover, the local application enables avoidance of first-pass metabolism in the liver, gastric pH variation, and fluctuations in plasma concentrations [29].

The aim of this work was to propose a new hydrogel based on methylcellulose containing suspended Bu particles, as an API, for intra-articular injections, which may ensure the prolonged release of Bu, longer anesthesia, and reduced injection frequency. The purpose of this work was also to reveal the influence of the acceptor medium composition on the release kinetics of Bu, in variable acceptor compartments, which reflected the viscosity and pH conditions in healthy and inflamed joints.

## 2. Materials and Methods

Bupivacaine (Bu) was delivered from Chemat (Gdańsk, Poland). Methylcellulose (MC) with the viscosity of 4000 cP was bought from Sigma-Aldrich (Steinhelm, Germany). Potassium dihydrogen phosphate, sodium hydroxide, hydrochloric acid 35–38%, and sodium phosphate dodecahydrate were obtained from Chempur (Piekary Śląskie, Poland). High-molecular-weight hyaluronic acid sodium salt (HAH > 1.1 MDa) and low-molecular-weight hyaluronic acid sodium salt (HAL = 4.5 kDa) were obtained from Esent (Szczecin, Poland). The cellulose membrane was purchased from Carl Roth (Karlsruhe, Germany). All chemicals were used without further purification.

### 2.1. Hydrogel Preparation

The preparation was obtained by mixing Bu as a medical substance, MC as the carrier, and water using the homogenizer (Unidrive X1000D, CAT Scientific Paso Robles, CA, USA) with the rotation speed of 16,000 rpm within 10 min. The concentration of the synthetic polymer was 2% (*w*/*w*). The concentration of the drug was also 2% (*w*/*w*). The hydrogel was stored at the temperature of 6 °C for 24 h in order to remove the air bubbles.

### 2.2. Acceptor Fluid Preparation

Phosphate buffers (PBS) at pH = 7.4 and pH = 6.8 were drawn up according to European Pharmacopoeia 10.8 [30]. The buffer at pH 7.4 was named PBS7.4 and the buffer at pH = 6.8 was tagged PBS6.8. The fluid PBS7.4 reflected the pH of normal synovial fluid (nSF) and the medium PBS6.8 corresponded to the pH of inflamed synovial fluid (iSF). HAH was added to the PBS7.4 buffer to obtain the concentration of HAH corresponding to the concentration of HAH in nSF. According to the literature data, it was 2.2 mg/mL [3]. This fluid was named PBS7.4H. HAL was added to the PBS6.8 medium to obtain a concentration the same as that of HAH in the PBS7.4H fluid. The achieved fluid with pH = 6.8 and containing HAL was called PBS6.8L and it was similar to the iSF. HAL was also added to the PBS7.4 buffer to obtain the PBS7.4L fluid and the concentration of HAL was the same as in the PBS6.8L medium. The compositions of the obtained acceptor fluids are summarized in Table 1.

### 2.3. Viscosity Study

The viscosity study of the prepared liquids PBS7.4, PBS6.8, PBS7.4H, PBS6.8L, and PBS7.4L were carried out employing the Ostwald viscometer. The flow times of the tested liquids and water, as the reference liquid, through the capillary of the viscometer were measured three times. The densities of the obtained fluids, as well as the density of water as the standard fluid, were measured employing the dedicated set for determining the density of liquids (WX-001-0001 with analytical balance AS X2 PLUS, Radwag, Radom, Poland). Three replicates were performed for each sample. The relative viscosity $\eta_{rel}$ was calculated from the following equation:

(1) $$\eta_{rel}=\frac{\eta_{s}}{\eta_{0}}=\frac{\rho_{s}\cdot t_{s}}{\rho_{0}\cdot t_{0}}$$

where $\eta_{s}$ and $\eta_{0}$ are the viscosity of the sample and the reference liquid (water), $\rho_{s}$ and $\rho_{0}$ are the density of the sample and the reference fluid (water), and $t_{s}$ and $t_{0}$ indicate the flow time through the viscometer capillary of the tested sample and the standard liquid (water). All measurements were performed at room temperature (about 25 °C).

### 2.4. UV–Vis Spectra of Bupivacaine and Calibration Curves

The UV–Vis spectra of Bu in the PBS7.4, PBS6.8, PBS7.4H, and PBS6.8L fluids were recorded using the spectrophotometer UV–Vis (Jasco V-530, Tokyo, Japan) at the speed of 400 nm/min in the wavelength range of 200–900 nm and at the resolution of 1 nm. The concentration of Bu in each medium was 0.2175 ± 0.0032 mg/mL. The calibration curves of Bu in the PBS7.4, PBS6.8, PBS7.4H, and PBS6.8L fluids were obtained at the wavelength corresponding to the maximum of absorbance. Based on these curves, the amount of the drug released to the acceptor fluid was calculated.

### 2.5. Release Study

The release of Bu from the hydrogel was carried out employing the USP Apparatus 4 (flow-through cell). This equipment, slightly modified, was described in detail in our previous works [31,32]. The flow cell was placed in a water jacket connected to an external thermostat (Prüfgeräte-Werk, B2 E10, Medingen, Germany) ensuring a constant measurement temperature, which was set at 37.0 ± 0.5 °C. The flow cell was placed on a magnetic stirrer (2mag magnetic ^e^motion, Muenchen, Germany) maintaining a constant mixing of the acceptor medium at a speed of 100 rpm. The cellulose membrane was placed on the upper part of the flow cell and a donor chamber, filled with the tested hydrogel, was placed on it. The flow of the acceptor fluid through the cell connected to the measuring cuvette in the spectrophotometer was provided by a peristaltic pump (PS-16 Sipper Pump, PG Instruments Limited, Leicestershire, UK) operating at a speed of 50 rpm. The medicinal substance was released to PBS7.4, PBS6.8, PBS7.4H, and PBS6.8L as the acceptor fluid. The absorbance of the acceptor medium was read every 2 min for 24 h. Based on the calibration curves, the amount of the drug released was calculated. The measurements were repeated six times for each acceptor fluid.

### 2.6. Difference Factor and Similarity Factor

According to FDA regulations [33], the difference factor (f_1_) and the similarity factor (f_2_) were determined based on the Formulas (2) and (3) presented below:

(2) $$f_{1}=\frac{\sum_{t=1}^{n}\left|R_{t}-T_{t}\right|}{\sum_{t=1}^{n}R_{t}}\times 100$$

(3) $$f_{2}=50\times log\left\{{\left[1+\frac{\sum_{t=1}^{n}{\left(R_{t}-T_{t}\right)}^{2}}{n}\right]}^{-0.5}\times 100\right\}$$

where n is the number of time points, R_t_ is the dissolution value of the reference batch at time t, and T_t_ is the dissolution value of the test batch at time t. The calculated values of coefficients f_1_ and f_2_ allowed for a comparison of the curves of the dependence of the Bu amount released over time.

### 2.7. Kinetic Study

The kinetics of the Bu dissolution from the prepared hydrogel to the PBS7.4, PBS6.8, PBS7.4H, and PBS6.8L acceptor fluids was analyzed using zero-, first-, and second-order equations as well as the Higuchi and Korsmeyer–Peppas models, which were presented in our previous works [32,34].

Based on these dependences, the physicochemical parameters such as the release rate constants (k) and the half-release time (t_0.5_) were calculated. Additionally, the parameter n from the Korsmeyer–Peppas model was also obtained.

### 2.8. Statistical Analysis

The obtained results were presented as arithmetic mean ± SD (standard deviation). The relative viscosity uncertainty was calculated by the total differential method. The comparison between the dissolution profiles of Bu from the hydrogel to the acceptor fluids PBS7.4, PBS6.8, PBS7.4H, and PBS6.8L was performed also employing the Student’s *t*-test at 95% confidence level. Although this statistical method is not approved by the FDA, it is useful in determining an improved understanding of the release data. The influence of pH on the Bu release from the formulation was analyzed by the comparison of the Bu release to PBS7.4 and to PBS6.8; the presence of HA in the liquid was not analyzed. The effect of the introduction of HMWHA into the medium was studied by the comparison of the Bu release into PBS7.4 or into PBS 7.4H; the pH was constant. The effect of introducing LMWHA into the acceptor fluid was studied by comparison of the Bu release to PBS6.8 or to PBS6.8L; the pH was constant. Differences between the Bu release into acceptor liquids reflecting the healthy and inflamed synovial fluid were analyzed by the comparison of the following: (1) the drug release into the acceptor medium of PBS6.8L to (2) the drug release into the acceptor medium PBS7.4H.

The kinetic analysis was carried out using the least squares method and calculating the correlation coefficient of determination R^2^. Based on the derived values of the correlation coefficients, the model that best described the release data was evaluated [35].

### 2.9. FTIR Measurements

The FTIR (Fourier Transform Infrared) spectra of pure MC, pure Bu, the physical mixture of MC and Bu, and the dried hydrogel composed of MC and water, as well as the dried formulation containing MC, Bu, and water, were recorded employing the FTIR spectrometer with ATR mode (Nicolet iS50, Thermo Scientific, Waltham, MA, USA). A total of 32 scans of each sample were collected with the speed of 65 scans per 1 min at the resolution of 16 cm^−1^. The measurements were carried out at room temperature and in the wavenumber range from 4000 cm^−1^ to 400 cm^−1^.

### 2.10. DSC Measurements

The thermograms of pure MC, pure Bu, the physical mixture of MC and Bu, and the dried preparation contained MC, Bu, and water were obtained using the DSC (Differential Scanning Calorimeter) calorimeter (214 Polyma, Netzsch, Wittelsbacherstraße, Germany). The thermal analysis was performed in the temperature range from −10 °C to 300 °C. The aluminum crucibles containing 3–5 mg of powder were closed with a lid, pressed, and placed in the device. The study were conducted under nitrogen atmosphere with a flow rate of 25 mL/min.

## 3. Results and Discussion

### 3.1. Viscosity Study

**Figure 3:**
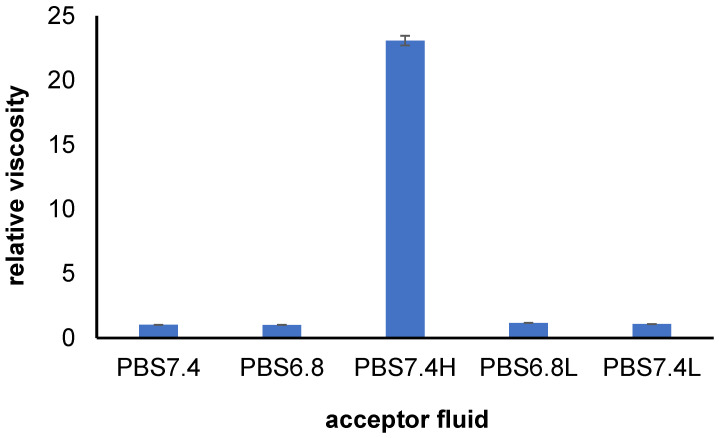
The obtained relative viscosity $\eta_{rel}=\frac{\eta_{s}}{\eta_{0}}$ of the acceptor fluids at room temperature; $\eta_{s}$ and $\eta_{0}$ are the viscosities of the sample and the reference liquid (water); n = 6, the error bars are the SD (standard deviation).

The obtained relative viscosity $\eta_{rel}$ of the employed acceptor media is presented in Figure 3. It was found that the relative viscosity of the liquids PBS7.4, PBS6.8, PBS6.8L, and PBS7.4L were close and were in the range from 1.02 to 1.18. However, the viscosity of the PBS7.4H fluid was much higher in comparison to the others and it was 23.09. This indicated that the incorporation of HAH to the medium influenced its viscosity. The incorporation of HAL did not affect the viscosity of the fluid; the viscosity of PBS6.8 and PBS6.8L were similar, and the viscosity of PBS7.4 and PBS7.4L were also comparable, meaning that neither pH nor HAL changed the viscosity of the media.

The increase in the viscosity of the PBS7.4H fluid was connected with the structure of HAH. It was found that the high-molecular-weight HA forms hydrogen bonds between the adjoined molecules as well as within one molecule. Additionally, the water molecule can form a bond between functional groups belonging to neighboring units. As a consequence, the primary structure of HA can be transformed into a secondary or even tertiary structure. Moreover, sodium salts of HAH have the double helix structure. Finally, high-molecular-weight HA creates twisted networks, capable of absorbing large amounts of water, which results in the formation of a large volume of chains of HA. This was manifested by the creation of high-viscosity hydrogels. The low-molecular-weight HA and solutions with a low concentration of HA are not capable of building the polymer network because the forces of the intermolecular interaction are very weak. HAL (about 10^3^ Da) at a low concentration provides an isolation of the macromolecules in the dilutant. Solutions of HA have properties of pseudoplastic liquids similar to Newtonian fluids. It was found that the relaxation time, which is the period from viscous properties to elastic properties, is lower in the case of HAH. The reduction in the HA chain length shortens the time needed to unravel the three-dimensional polymer network [9,36].

The differences in viscoelastic properties of HA depending on the molecular weight of the biopolymer are important in the treatment of joint diseases. It was found that the rheological properties of the synovial fluid change when the joint becomes inflamed. HAH with a molecular weight of 6.3–7.6 MDa and a concentration of 2.5–4.0 mg/mL is present in healthy synovial fluid. However, in the case of inflamed synovial fluid, the molecular weight of HA decreases to 1.6–3.48 MDa and the concentration of this biopolymer is reduced to 1.0–2.0 mg/mL [27].

### 3.2. Release Study

**Figure 4:**
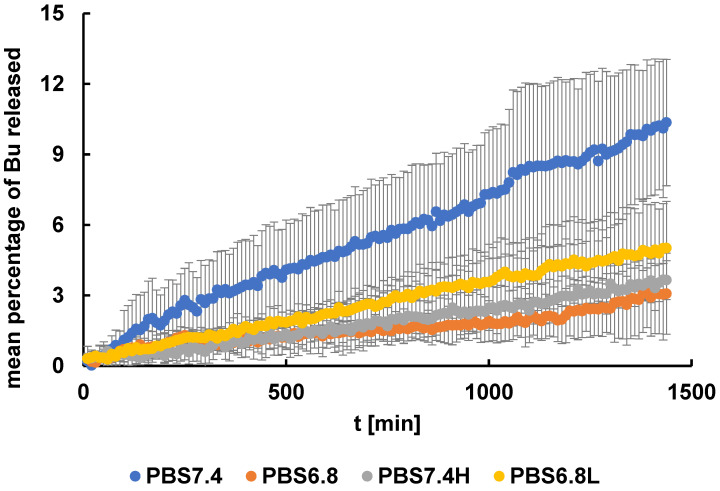
The profiles of Bu released from the formulation studied to the acceptor fluids PBS7.4, PBS6.8, PBS7.4H, PBS6.8L; n = 6, the error bars are the SD (standard deviation).

The mean percentages of Bu released from the formulation studied to the acceptor fluids PBS7.4, PBS6.8, PBS7.4H, and PBS6.8L are shown in Figure 4.

It was observed that the smallest amount of the drug was released from the gel within 24 h into the liquid PBS6.8 and PBS7.4H and it was 3.1 ± 1.7% and 3.7 ± 0.8%, respectively. In the case of using the acceptor fluid PBS6.8L, it was 5.0 ± 2.0%. The highest amount of Bu, 10.4 ± 2.7%, was released into the fluid PBS7.4. It can be summarized that within 24 h, the total amount of Bu was not released from the hydrogel either into the fluid representing the normal synovial fluid (PBS7.4H) or into the model medium corresponding to the inflamed synovial fluid (PBS6.8L). In addition, all Bu release curves did not show a characteristic plateau, a constant concentration of the drug in the acceptor fluid that does not change over time. The obtained results may therefore suggest that the proposed formulation may be an intra-articular sustained release preparation.

### 3.3. Release Curves Comparison

The calculated values of the difference factor f_1_ and the similarity factor f_2_ obtained from the evaluation of the release curves of Bu to the acceptor fluids PBS7.4, PBS6.8, PBS7.4H, and PBS6.8L are presented in Table 2. In all comparisons, the difference factors were above 15, indicating the discrepancy between the dissolution curves. However, in the case of the similarity factors, the values were below 50, meaning no differences were found between the release patterns. It can be concluded that the analysis based on the determination of the f_1_ and f_2_ parameters, recommended by the FDA, did not give the unequivocal answer whether there were differences between the obtained release curves of Bu to the used acceptor media. For this reason, in the next stage of the research, a statistical analysis was carried out using the Student’s *t*-test. The studies showed that there were statistically significant differences between all the compared curves.

The discrepancies between the release curves of Bu into the liquids PBS7.4 and PBS6.8 indicated that the pH of the medium affected the release of the drug from the formulation. It may be caused by differences in the solubility of Bu in solutions with different pH levels. According to numerous studies, it was revealed that the solubility of a substance in the diffusion layer determines the intrinsic dissolution rate, not the solubility in the acceptor medium [37,38,39]. Additionally, the pH of the acceptor fluid is different than the pH of the diffusion layer. Shah et al. [39] found that the pH of the diffusion layer was 6.6 when the pH of the acceptor fluid was 3.0 or 5.0, although when the pH of the liquid was 7.4, the pH of the diffusion layer was 7.6. This suggested that when the value of the pH of the acceptor fluid used in the present research was 6.8, the value of the pH in the diffusion layer was higher and could even change from slightly acidic to slightly alkaline. This was connected with the self-buffering effect. In an environment where Bu is in an ionized form, the amino group can attach protons, causing an increase in pH, although in an alkaline environment, bupivacaine does not exist in the form of ions, so the increase in pH is insignificant. The solubility of the drug in the diffusion layer determined the driving force for the release. The microenvironment was important at the solid–liquid interface, not within the entire medium [39].

The pKa value 8.2 of Bu, the weak base, suggests a higher dissolution rate and consequently a higher release rate into a slightly acidic environment, e.g., of pH 6.8, when compared to a slightly alkaline environment of pH 7.4, where the dissolution and release rates should be lower [39]. However, the experimental data of the release of Bu into these solutions, as assessed in the present work, and shown in Figure 4, indicated a reverse situation. The apparently inconsistent release of Bu observed in our studies may be a result of Bu being enclosed in a viscous hydrogel matrix. Additional effects may include the formation of diffusion barriers, hydration shells, and micelles and the penetration of buffer components into the matrix. The presence of HA salt also plays an important role, which certainly affects the presence of ions in the water system. It may be concluded that the presence of a diffusion layer and other components of the system significantly affected the Bu transport into the acceptor fluid.

The comparison of the release profiles of Bu to the PBS6.8 and PBS6.8L fluids revealed that the introduction of HAL influenced the release of the drug from the gel. The relative viscosities of the PBS6.8 and PBS6.8L liquids were similar and only the presence of HAL affected the increase in the dissolution rate. The pKa values of the un-ionized and the ionized forms of Bu estimated by Shah et al. [39] were similar and were 8.24. The polysaccharide chain is negatively charged with pKa between 3.0 and 4.0 [40]. This may suggest that the short polymer chains interact electrostatically with Bu and accelerate the drug transportation from the formulation to the acceptor medium.

Changes in the pattern of Bu release curves into the PBS7.4 and PBS7.4H fluids were also observed, when the HAH was incorporated into the acceptor fluid. However, the Bu dissolution into PBS7.4 and PBS7.4H gave reverse results than in the case of using the PBS6.8 and PBS6.8L media. The differences in the Bu release pattern to PBS7.4 and PBS7.4H may be caused mainly by the discrepancies in the viscosity of both liquids. The incorporation of HAH increased the viscosity of the medium (Figure 2) resulting in the drug dissolution reduction. According to Hassan et al. [41], who studied naproxen release from tablets, the decrease in the drug dissolution with the increased acceptor fluid viscosity was caused by the formation of the polymeric coat layer surrounding the formulation. The created barrier blocked the solvent’s access to the interior with the drug. Additionally, it was postulated that the hydrophilic polymers with high molecular weights may significantly influence the drug release by forming a hydrate layer in the hydrophilic medium. In the present work, the polymer incorporated to the fluid, which was HAH, was also hydrophilic with high molecular weight, explaining the differences in the Bu release to PBS7.4 and PBS7.4H. An inconsistency was also found between the release curves of Bu from the formulation to PBS7.4H and to PBS6.8L that can be explained by the same process mentioned above. This is very important in joint therapy, because the acceptor medium PBS7.4H reflected the normal synovial fluid and the PBS6.8L liquid imitated the synovial fluid of the inflamed joint. The obtained results may suggest that Bu was released differently to the normal synovial fluid in comparison to its release to the inflamed synovial fluid.

### 3.4. Kinetic Analysis

**Figure 5:**
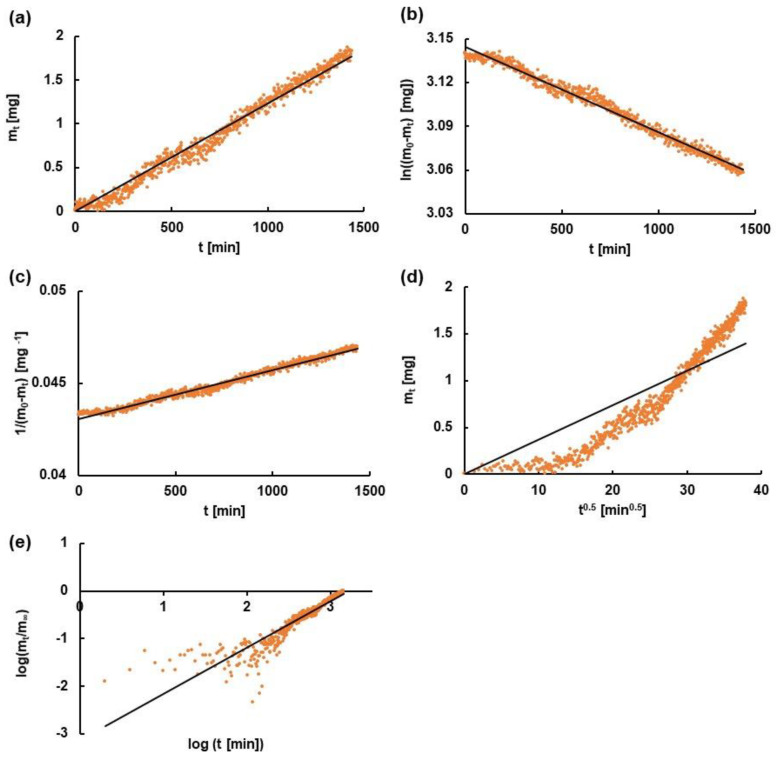
The fitting of experimental points (dots) to theoretical curves (lines) based on (**a**) zero-, (**b**) first-, and (**c**) second-order equations and on (**d**) Higuchi and (**e**) Korsmeyer-Peppas models, on the example of Bu release to the acceptor fluid PBS6.8L; m_t_—the amount of the drug released in time t; m_0_—the amount of the drug in the formulation before the dissolution; m_∞_—the amount of the drug released after infinitive time.

The kinetic analysis of the Bu release from the preparation to the media PBS7.4, PBS6.8, PBS7.4H, and PBS6.8L was performed based on zero-, first-, second-order equations as well as the Higuchi and Korsmeyer–Peppas models. The example fitting of experimental points to theoretical curves based on the employed kinetic models is presented in Figure 5. The achieved kinetic data from this study are listed in Table 3.

It was found that the models that best described the Bu release from the formulation to the acceptor fluids PBS7.4, PBS6.8, PBS7.4H, and PBS6.8L were the first-order kinetics and the second-order equation. It was noticed that the release rate constant was the highest when the drug was released to the medium PBS7.4. The half-release time was the lowest when the drug was transported from the hydrogel to the liquid PBS7.4, meaning that this was the fastest process. The addition of HAH to PBS7.4 decreased the values of k_1_ and k_2_, calculated from models that described the studied process the best, indicating the slowing down of the dissolution process. However, the incorporation of HAL into the acceptor fluid with pH = 6.8 increased the release rate constants k_1_, k_2_. The chain length of HA as well as the pH affected the release kinetics of the drug from the hydrogel.

### 3.5. FTIR Study

**Figure 6:**
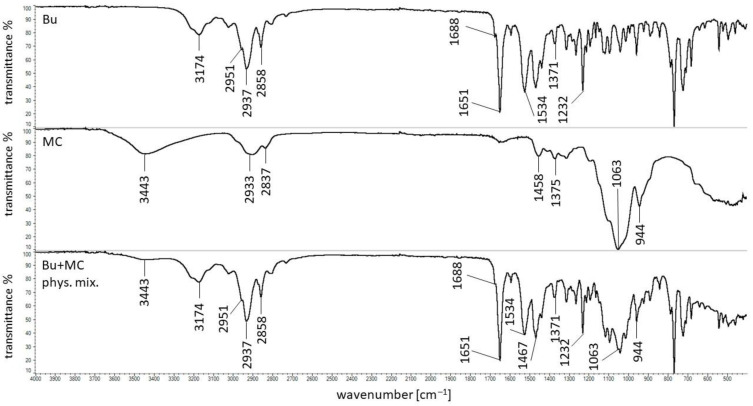
The FTIR spectra of pure Bu, pure MC, and the physical mixture of Bu and MC.

The FTIR spectra of Bu, MC, and their physical mixture are shown in Figure 6. The following characteristic bands of Bu were observed: at 3174 cm^−1^ coming from the stretching of the hydrogen-bonded N-H group of the mono-substituted amides O=C-N-H; at 2937 cm^−1^ corresponding to CH_3_ stretching vibrations; at 1688 cm^−1^ related to the stretching vibrations of C=C; and at 1651 cm^−1^ assigned to the C=O stretching vibrations of amide I group. Additionally, the signals at 1534 cm^−1^ coming from the amide II vibration, at 1371 cm^−1^ assigned to CH_3_ bending, and at 1232 cm^−1^ related to C-N-H stretch vibrations were also found and all the maxima were consistent with the data from previous works [42,43,44,45].

In the FTIR spectrum of pure MC, the maximum at 3443 cm^−1^ was observed, which was assigned to the stretching vibration of the –OH group. The signals at 2933 cm^−1^ and 2837 cm^−1^ were related to the asymmetric and symmetric stretching vibrations of C-H, respectively. The bands at 1458 cm^−1^ and 1375 cm^−1^ corresponded with the vibration of the deformation in the plane of δ (C-H). The peak at 1063 cm^−1^ was attributed to the C-O-C stretching mode. The band at 944 cm^−1^ was assigned to the OCH_3_ group. The recorded FTIR spectrum of MC was consistent with that presented by Nadour et al. [46].

All the characteristic bands of Bu were found in the FTIR spectrum of the physical mixture of the ingredients (Bu and MC). However, the signals of MC at 2933 cm^−1^ and 2837 cm^−1^ were invisible because they were covered by strong Bu signals at 2937 cm^−1^ and 2858 cm^−1^. Additionally, the weak MC maximum at 1458 cm^−1^ was overlapped by a stronger peak of Bu at 1467 cm^−1^ and the MC band at 1375 cm^−1^ was also covered by the peak of Bu at 1371 cm^−1^. The MC bands at 1063 cm^−1^ and 944 cm^−1^ were observed. The presence of all maxima of Bu and MC in the FTIR spectra of the components’ physical mixture indicated that there was no interaction between the ingredients of the mixture.

**Figure 7:**
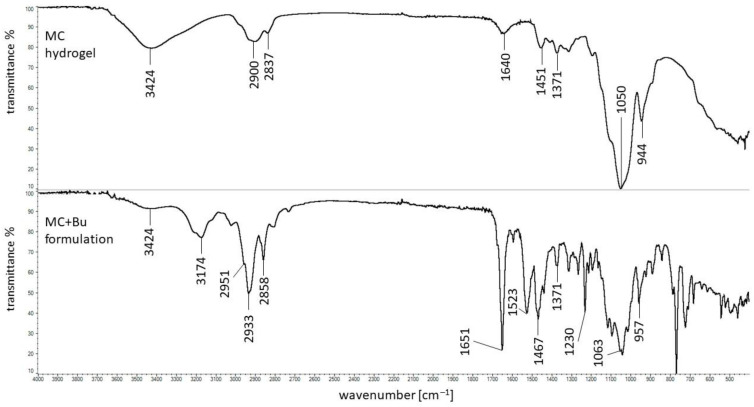
The FTIR spectra of the dried MC hydrogel and the dried formulation composed of Bu, MC, and water.

The FTIR spectra of the dried MC hydrogel and the dried formulation composed of Bu, MC, and water are presented in Figure 7. It was revealed that all characteristic bands assigned to the presence of MC were observed in the spectrum of the MC hydrogel. However, this spectrum may be disturbed by the presence of water. This molecule gives two intense broad bands at 3500 cm^−1^ and 1635 cm^−1^ which were assigned to the O-H stretching and the O-H-O scissor-bending, respectively [47]. In the spectrum of the MC-based formulation with Bu, all the characteristic bands of Bu and MC were found, although the broad band at 3424 cm^−1^ may come from the water molecule. This spectrum is similar to the spectrum of the physical mixture of these compounds suggesting no interaction between the formulation ingredients.

### 3.6. DSC Investigations

**Figure 8:**
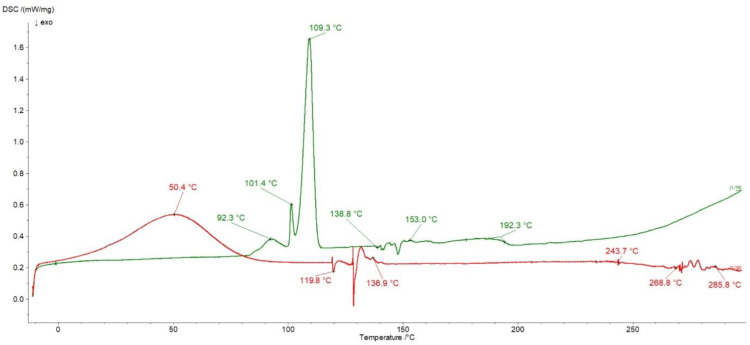
Thermograms of Bu (green), MC (red).

The thermograms of pure Bu and MC are presented in Figure 8. In the heating curve of Bu, three sharp endotherms at 92.3 °C, 101.4 °C, and 109.3 °C occurred and they were connected with a dehydration process [43,48,49]. The peaks from 138.8 °C to 153.0 °C corresponded to the metastable form of Bu that changed to the stable form existing in the temperature range of 190–200 °C [49].

**Figure 9:**
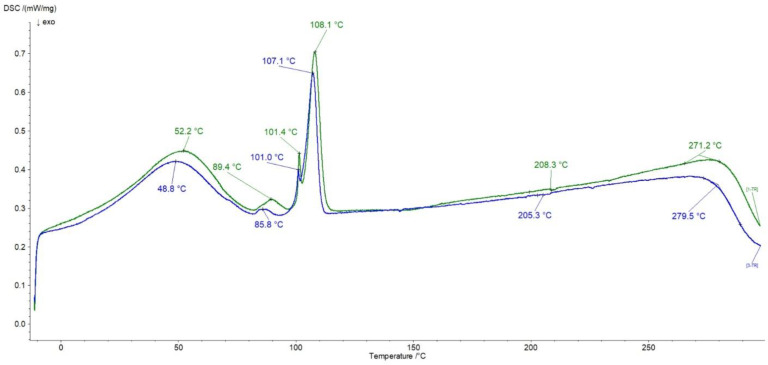
Thermograms of the physical mixture of Bu and MC (blue), and the formulation composed of Bu, MC, and water (green).

In the thermogram of MC a broad endotherm was observed with the maximum at 50.4 °C attributed to water evaporation. According to Nadour et al. [46], the signals between 119.8 °C and 136.9 °C were assigned to the glass transition (Tg). The Tg values of MC from different literature data may be varied because methylcellulose samples may differ in the degree of substitution of methyl groups, a high water content in the sample may lower the Tg, and the origin of the cellulose from which the derivative was obtained may also affect the Tg value [50]. The peaks at 243.7 °C and the series of signals from 268.8 °C to 285.8 °C were attributed to the decomposition of the sample [50]. All the mentioned maxima of Bu and MC, apart from the Tg signal from MC, were present in the thermogram of the physical mixture of Bu and MC presented in Figure 9.

In the case of the formulation composed of Bu, MC, and water, the endotherms attributed to the Bu dehydration process at 89.4 °C, 101.4 °C, and 108.1 °C, as well as the endotherm coming from the water lost by MC at 52.2 °C, were observed (Figure 9). The weak peak at 208.3 °C assigned to the Bu decomposition was found and the broad maximum at 271.2 °C related to the polymer decomposition was noticed. It should be mentioned that the thermogram of the physical mixture of Bu overlapped with the heating curve of the formulation meaning that there were no interactions between ingredients.

## 4. Conclusions

The presented investigation revealed that the release pattern of Bu from the hydrogel depended on the acceptor fluid. The composition of the acceptor medium, its pH, and the viscosity influenced the Bu release kinetics. A high release rate was observed in the case of the acceptor fluid of pH = 7.4; however, the addition of HAH to this medium remarkably increased its viscosity and resulted in decreased Bu release. The release of Bu into the acceptor fluids, representing the synovial fluid of a healthy joint or the synovial fluid of an inflamed joint, had the opposite pattern than the release of Bu into the buffers of pH = 7.4 and pH = 6.8 not doped with HA. A higher Bu release rate for fluids doped with HA was observed for pH = 6.8. No interactions between the formulation ingredients in the dry state was found; however, the results indicate possible ionic interactions in the aqueous phase, between the ingredients of the formulation and components of the acceptor fluids, which could interfere with drug release.

## Figures and Tables

**Table 1:** The composition of the obtained acceptor fluids (PBS means phosphate buffer, HA is sodium hyaluronate).

Fluid	PBS7.4	PBS6.8	PBS7.4H	PBS6.8L	PBS7.4L
pH of PBS	7.4	6.8	7.4	6.8	7.4
HA	—	—	High molecular	Low molecular	Low molecular

**Table 2:** The calculated values of the difference factor (f_1_) and the similarity factor (f_2_) obtained from the comparison of the release curves of Bu to the acceptor fluids PBS7.4, PBS6.8, PBS7.4H, PBS6.8L.

Fluid	PBS6.8		PBS7.4H	
	f_1_	f_2_	f_1_	f_2_
PBS7.4	71.4	67.0	66.7	68.9
PBS6.8L	71.7	88.8	46.0	93.0

**Table 3:** The calculated kinetic parameters of the Bu release from the formulation to the evaluated acceptor fluids PBS7.4, PBS6.8, PBS7.4H, PBS6.8L.

Kinetic Model	Kinetic Parameters	PBS7.4	PBS6.8	PBS7.4H	PBS6.8L
Z-O	k_0_ × 10^3^ [mg × min^−1^]	1.91 ± 0.02	0.51 ± 0.01	0.63 ± 0.01	0.90 ± 0.01
	t_0.5_ [min]	7532 ± 64	28,666 ± 828	21,650 ± 340	15,443 ± 172
	R^2^	0.94 ± 0.03	0.53 ± 0.27	0.86 ± 0.06	0.96 ± 0.02
F-O	k_1_ × 10^5^ [min^−1^]	7.20 ± 0.01	1.60 ± 0.07	2.36 ± 0.06	3.5 ± 0.05
	t_0.5_ [min]	10,561 ± 156	59,002 ± 4380	30,411 ± 860	26,653 ± 546
	R^2^	0.96 ± 0.01	0.64 ± 0.23	0.88 ± 0.07	0.95 ± 0.05
S-O	k_2_ × 10^6^ [mg^−1^ × min^−1^]	3.00 ± 0.04	0.67 ± 0.003	0.96 ± 0.03	1.45 ± 0.02
	t_0.5_ [min]	14,468 ± 217	84,016 ± 6258	43,072 ± 1220	37,577 ± 771
	R^2^	0.96 ± 0.01	0.63 ± 0.23	0.88 ± 0.07	0.95 ± 0.05
H	k_H_ × 10^2^ [mg × min^−1/2^]	5.85 ± 0.08	1.57 ± 0.03	1.93 ± 0.03	2.74 ± 0.04
	t_0.5_ [min]	66,471 ± 2109	969,656 ± 51,061	548,209 ± 26,166	272,192 ± 6665
	R^2^	0.84 ± 0.07	0.60 ± 0.16	0.75 ± 0.15	0.83 ± 0.04
K-P	k_K-P_ × 10^3^ [min^−n^]	3.6 ± 0.2	25.9 ± 2.5	5.2 ± 0.6	16.0 ± 0.6
	t_0.5_ [min]	712 ± 73	810 ± 190	848 ± 169	615 ± 57
	R^2^	0.87 ± 0.06	0.47 ± 0.21	0.62 ± 0.16	0.88 ± 0.03
	n	0.82 ± 0.02	0.54 ± 0.04	0.70 ± 0.03	0.75 ± 0.02
best fit		F-O, S-O	F-O, S-O	F-O, S-O	Z-O, F-O, S-O

Z-O—zero-order model; F-O—first-order model, S-O—second-order model; H—Higuchi model; K-P—Korsmeyer–Peppas model; k_0_—the zero-order release rate constant; k_1_—the first-order release rate constant; k_2_—the second-order release rate constant; k_H_—the Higuchi rate constant; k_K-P_—the Korsmeyer–Peppas rate constant, n—the parameter indicative of the drug release mechanism, t_0.5_—the half-release time, R^2^—correlation coefficient.

## Data Availability

The original contributions presented in this study are included in the article; further inquiries can be directed to the corresponding author. The data are not publicly available due to high number of repeated values of consecutive measurements.
